# Phylogenetic Revisit to a Review on Predatory Bacteria

**DOI:** 10.3390/microorganisms11071673

**Published:** 2023-06-27

**Authors:** Saki Kamada, Ryoka Wakabayashi, Takeshi Naganuma

**Affiliations:** Graduate School of Integrated Sciences for Life, Hiroshima University, 1-4-4 Kagamiyama, Higashihiroshima 739-8528, Japan

**Keywords:** bacterial predation, *Bdellovibrionota*, BALOs, *Myxococcota*, predatome

## Abstract

Predatory bacteria, along with the biology of their predatory behavior, have attracted interest in terms of their ecological significance and industrial applications, a trend that has been even more pronounced since the comprehensive review in 2016. This mini-review does not cover research trends, such as the role of outer membrane vesicles in myxobacterial predation, but provides an overview of the classification and newly described taxa of predatory bacteria since 2016, particularly with regard to phylogenetic aspects. Among them, it is noteworthy that in 2020 there was a major phylogenetic reorganization that the taxa hosting *Bdellovibrio* and *Myxococcus*, formerly classified as *Deltaproteobacteria*, were proposed as the new phyla *Bdellovibrionota* and *Myxococcota*, respectively. Predatory bacteria have been reported from other phyla, especially from the candidate divisions. Predatory bacteria that prey on cyanobacteria and predatory cyanobacteria that prey on *Chlorella* have also been found. These are also covered in this mini-review, and trans-phylum phylogenetic trees are presented.

## 1. Introduction

As comprehensively reviewed by Pérez et al. (2016) [1], predatory bacteria are a group of prokaryotes that can actively hunt and consume other bacteria as their food source. By doing so, they can alter the abundance and diversity of the prey bacteria and thus influence the overall structure of the microbial community. In addition to predatory bacteria, protists and bacteriophages can also have significant impacts on the biomass, structure, and function of microbial communities, though their impacts differ in size, prey specificity, and hunting tactics [2]. Among their interwoven interactions, this mini-review, as an update of Pérez et al. (2016) [1], focuses on predatory bacteria with reference to phylogenetic aspects, particularly after the proposal in 2020 of the new phyla *Bdellovibrionota* and *Myxococcota*, which show distinct hunting strategies of predation [3].

Pérez et al. (2016) [1] reviewed the hunting strategies of predators of the order *Bdellovibrionales,* which physically attach to prey cells with flagella-based motility and penetrate into the periplasm of the prey cells, and the order *Myxococcales*, which are known for a “group attack” with gliding motility, the secretion of lytic enzymes, and the release of antibiotics. Pérez et al. (2016) [1] also reviewed the genomes, transcriptomes, and comparative genomics of predators, including the idea of the “predatome”, i.e., the protein families in phenotypes of predatory bacteria [4]. Through detailed analysis of the predation-related proteins and the encoding genes, predatory properties are predicted for the clades whose predations are not yet known in the phyla *Bdellovibrionota* and *Myxococcota* [3]. Moreover, detailed analyses on the correlation between antibiotics biosynthesis and predation indicate that myxobacteria may be prioritized for the discovery of unexplored natural products [5,6,7].

After Pérez et al. (2016) [1], ecological significances and industrial applications of predatory bacteria have been increasingly studied. For example, a study on the potential use of predatory bacteria as alternatives to antibiotics showed that intrarectal inoculations of *Bdellovibrio bacteriovorus* and *Micavibrio aeruginosavorus* lead to beneficial and adverse changes, respectively, in rat gut microflora, indicating a top-down control [8]. A large-scale field study using stable isotopes ^18^O and ^13^C demonstrated that activities of obligate predators are increased by substrates added to preys, indicating a bottom-up trophic control [9]. A high-resolution microscopic study revealed the submillimeter-scale changes in *Vibrio cholerae* biofilms attacked by *Bdellovibrio bacteriovorus* [10]. A recent review evaluates that potential uses of *Bdellovibrio* and like organisms (BALOs) in medical, agricultural, biotechnological, and environmental applications are achievable and should be pursued [11].

This mini-review aims to update Pérez et al. (2016) [1] based on these publications along with previous ones and to present a comprehensive, trans-phylum phylogenetic tree of predatory bacteria. A PubMed search using the simple phrase “predatory bacteria” (double quotations are needed to combine “predatory” and “bacteria” as a solid phrase) resulted in a total of 137 publications in and after 2016, with a peak of 24 hits in 2021 (accessed on 16 May 2023; https://pubmed.ncbi.nlm.nih.gov/?term=predatory+bacteria&filter=years.2016-2023). The number decreases to 21 from 137 when the search word “phylogenetics” is added. One of the 21 hits reports a genomically characterized nonpredatory nature of an isolate related to the predatory genus *Herpetosiphon* in the phylum *Chloroflexota* [12], which represents a possibility of a predatome-based search for predatory bacteria in diverse taxa.

## 2. Phylogenetics of Predatory Bacteria in and after 2016

### 2.1. Trans-Phylum Phylogenetics in and after 2016

Trans-phylum phylogenetic analysis of predatory bacteria was conducted in some of the PubMed-searched publications in and after 2016. In metagenomics of the intertidal soils along the Peruvian coastline, about 0.5% of 16S rRNA gene sequences are ascribed to predatory bacteria of multiple phyla, and 30 antibiotic-producing strains are kept in cultures with *Escherichia coli XL1* Blue or *Pheobacter inhibens* DSM17395 as prey [13]. In the V4–V5 microbiomics of the Chesapeake Bay sediment, amplicons are affiliated with the predatory genus *Haliangium* and the class *Polyangia* (phylum *Myxococcota*), the genus-level clade of the phylum *Bdellovibrionota*, and the *Bradymonadales* clade of the phylum *Desulfobacterota*, along with the abundant amplicons affiliated with *Ca. Electrothrix* of the phylum *Desulfobacteriota* [14]. A metatranscriptomic study combined with a set of curated genomes revealed increased and decreased expressions of proteases by the members of the phyla *Bacteroidota*-*Actinobacteriota* and *Myxococcota*, respectively [15].

### 2.2. Phylogenetics on the Members of BALOs, or the Phylum Bdellovibrionota, in and after 2016

Most of the PubMed-searched publications on predatory bacterial phylogenetics deal with selected taxa such as BALOs in the phylum *Bdellovibrionota*, for example, with a co-occurrence microbial network, particularly with *Myxococcales* of the phylum *Myxococcota* [16]. Two major BALO clades, i.e., the *Bacteriovorax* and *Bdellovibrio* clades, are targeted to decipher their roles and interplays in the microbiomes of wastewater treatment plants [17]. Comparative genomics of ten BALO genomes and one genome of a marine *Bacteoidota* predator *(Saprospira grandis*) allowed the genome-mining of 18 putative “predatomes” [18]. Comparative genomics of 152 BALO genomes from databases and 5 metagenome-assembled genomes (MAGs) from the Mariana Trench deep-sea water suggested that the chitinase-possessing members of *Oligoflexia*, the third class of the phylum *Bdellovibrionota*, may also act as predatory bacteria [19].

Phylogenetic aspects have also been viewed from the prey side. A wide prey range of a marine, prey-generalist BALO, *Halobacteriovorax marinus*, is discussed in evolutionary reference to horizontal gene transfer [20], although other *Halobacteriovorax* isolates (no phylogenetic information available) are rather prey-specific to *Vibrio parahaemolyticus*, a seafood-associated pathogen, and not to other seafood-associated pathogens, such as *Vibrio vulnificus*, *Vibrio alginolyticus*, *Escherichia coli* O157:H7, and *Salmonella enterica* serovar Typhimurium DT104 [21]. *Bdellovibrio bacteriovorus* is also known as prey-specific to Gram-negative bacteria by burrowing through the outer membrane and peptidoglycan cell wall and entering the periplasmic space of the prey cells [22].

Prey-specificity or prey-generality may be correlated to the alpha diversity of the BALO-associated microbiomes as suggested by a correlation study based on nine microbiomes from animals and environments [23], although this correlation is currently a correlation, not strong enough to be a prediction. Among the BALOs, alpha and beta diversities of the *Halobacteriovorax* clades in an estuary microbiome may be driven by inputs of pollutants, such as dissolved inorganic phosphorus and NH_4_^+^-N, as implicated by an analysis of the 676F-1193R region of the 16S rRNA gene sequence [24], though they are probably indirectly driven via bottom-up trophic control [9].

Detailed genotypic and phenotypic comparisons have also been performed for a soil isolate *Bdellovibrio* sp. NC01 and the type strain *Bdellovibrio bacteriovorus* HD100, indicating that reduced predatory activities of the isolate NC01 are probably due to the absence of ten genes in its genome [25].

### 2.3. Phylogenetics on the Members of the Predatory Phylum Myxococcota in and after 2016

A microbiomic study at a wastewater treatment plant using ^13^C-labeled *Escherichia coli* ESS5 and *Pseudomonas putida* ESE1 as preys discovered the dominance of *Haliangium* and the mle1–27 clade of the phylum *Myxococcota*, contrary to the conventional *Bdellovibrionota*-dominant view, which is further confirmed by analyzing the global datasets [26] from the Global Water Microbiome Consortium [27]. A large-scale study on agricultural soil microbiomes revealed that, out of all 6151 bacterial operational taxonomic units (OTUs), 242 are the myxobacterial OTUs affiliated with the phylum *Myxococcota* and that the alpha and beta diversities of the myxobacterial communities are more sensitive to geography (location and climate) than fertilization [28].

## 3. Description of New Taxa and Characterization of New Strains

### 3.1. Candidate Divisons

Co-cultured with the methanogenic archaeon, *Methanosaeta*, which is probably the globally prevalent methane producer [29], an epibiotic bacterial strain OP3 LiM that preys on the archaeon *Methanosaeta* has been characterized by genomics and proteomics as well as fluorescence and electron microscopy. The strain OP3 represents the candidate genus and a species, “*Ca*. *Velamenicoccus archaeovorus*” gen. nov., sp. nov., affiliated with the phylum “*Ca. Omnitrophica*” (candidate division OP3) [30].

Two new predatory species from formerly candidate divisions have also been reported. One is the bacterial strain TM7x HMT-952, affiliated with the formerly candidate division TM7 (currently the phylum “*Ca. Saccharibacteria*”). TM7x is epibiotic to *Actinomyces odontolyticus* subspecies *actinosynbacter* XH001 [31,32,33] and proposed to be designated as “*Ca*. *Nanosynbacter lyticus*” TM7x [34,35].

Another one is the ultra-small epibiont to the gammaproteobacterial photoautotroph *Chromatium minus*, inhabiting the karstic lakes in northeastern Spain, that was tentatively named *Vampirococcus* in 1986 [36]. In the same article, a Gram-negative, facultatively anaerobic epsilonproteobacterium, *Daptobacter*, was also mentioned but has been less studied and unvalidated. In 2013, *Chromatium (Halochromatium)*-like bacteria and associated ultra-small (550 nm × 220 nm) epibionts, which morphologically matched to the *Vampirococcus* reported in 1986, were sampled in a hypersaline lake in northeastern Spain and subject to a two-member consortium mini-metagenomic analysis, yielding a genome similar to the genomes affiliated with the superphylum “*Ca. Patescibacteria*” (the candidate phyla radiation, CPR) or “*Ca. Absconditabacteria*” (formerly SR1). Based on the putative *Vampirococcus* genome, “*Ca*. *Vampirococcus lugosii*” has been proposed [37].

### 3.2. Phyla Bdelovibrionota and Myxococcota

The 16S rRNA gene sequences of a soil bacterium strain LBG001 and the type strain *Bdellovibrio bacteriovorus* HD100 share a 97% similarity, which does not discriminate based on a traditional 97% threshold [38] but does discriminate based on an updated view [39]. The genomic features of LBG001, i.e., its average nucleotide identity, average amino acid identity, and digital DNA–DNA hybridization values with other *Bdellovibrio* members, are as low as <79%, <72% and <17%, respectively, enough to be discriminated, and thus LBG001 is described as the new species *Bdellovibrio reynosensis* [40], whose specific epithet indicates its place of origin, Reynosa, a Mexican city on the southern bank of the Rio Grande.

One of the five new species of the genus *Myxococcus* described in 2020 is also named after its place of origin, i.e., a settlement on the island of Anglesey in North Wales, UK. The settlement’s name appears in the specific epithet as *Myxococcus llanfairpwllgwyngyllgogerychwyrndrobwllllantysiliogogogochensis*, whose 16S rRNA gene sequence is not explicitly available from its shotgun-sequenced genome [41].

Two new predatory strains isolated from a freshwater pond, *Bacteriovorax stolpii* HI3 (phylum *Bdelovibrionota*) and *Myxococcus* sp. MH1 (phylum *Myxococcota*), show a wide range of preys, including *Escherichia coli* HB101 and 52 environmental strains consisting of 8 Gram-positive strains affiliated with the phyla *Actinomycetota* and *Bacillota* and 44 Gram-negative strains affiliated with the phyla *Bacteroidota* and *Pseudomonadota*. While *Myxococcus* sp. MH1 preys on all the 53 prey strains, *Bacteriovorax stolpii* HI3 preys on 25 environmental Gram-negative strains as well as *E. coli* HB101 [42].

The term “myxobacteria” (or “slime bacteria”) has been ambiguously defined but may be regarded as the members of the orders *Myxococcales* and *Polyangia* in the phylum *Myxococcota* [43]. Omics studies, including “predatomics” of myxobacteria based on 163 publicly available genomes and 24 newly added genomes, were reviewed in 2021 [44]. More extensive review on myxobacteria with >400 references has recently been published [43].

### 3.3. Phylum Planctomycetota

The phylum *Planctomycetota*, along with *Verrucomicrobiota* and *Chlamydiota*, composes the superphylum PVC, whose member is hypothesized to be an ancestor of the proto-eukaryotic cell via symbiogenesis with an archaeon [45,46]. Within the phylum *Planctomycetota*, a bacterium that exhibits eukaryote-like phagocytosis predation was discovered from the seawater of Palau. The bacterium is described as “*Ca*. *Uab amorphum*”, whose genus is named after a giant of Palauan mythology, indicating its size as giant (4.5–7.8 μm × 2.8–5.5 μm), and it is capable of engulfment or phagocytosis [47].

### 3.4. Order Bradymonadales in the Phylum Desulfobacteriota

In addition to the two major taxa of predatory bacteria, i.e., the phyla *Bdelovibrionota* and *Myxococcota*, the order *Bradymonadales* may represent a third type of predation. The order *Bradymonadales* was formerly ascribed to the class Deltaproteobacteria of the phylum Proteobacteria but is currently affiliated with the phylum *Desulfobacteriota* [3]. The bacteria of this order, or *Bradymonabacteria*, show a unique predation type that is different from the so-called “obligate” and “facultative” as defined in Pérez et al. (2016), [1] which are characterized as “completely prey-dependent” and “prey-independent” [48]. The predation type of *Bradymonabacteria* is characterized as “facultatively prey-dependent” and termed lately as “facultative” [48], and the previous term “facultative” from Pérez et al. (2016) [1] was newly replaced with “opportunistic”.

Within the order *Bradymonadales*, a new genus and new species has been proposed for the wide-ranging predatory strain YN101 from the sediment of a marine solar saltern, which is described as *Persicimonas caeni* gen. nov., sp. nov. in the family *Bradymonadaceae* [49].

Another new genus has also been proposed during the descriptions of two new species, *Lujinxingia litoralis* gen. nov. sp. nov. and *Lujinxingia sediminis* sp. nov., in the family *Bradymonadaceae* [50]. The genus name *Lujinxingia* is named after a Chinese microbiologist, Jin-Xing Lu, or Lu Jin-Xian in the Chinese name order.

A detailed comparison between *Lujinxingia sediminis* and a related strain, V1718, from a Chinese island, has led to the proposal of two new families, in addition to the family *Bradymonadaceae*, within the order *Bradymonadales*. During the description of the strain V1718 as *Microvenator marinus* gen. nov., sp. nov., two new families, *Microvenatoraceae* fam. nov. and *Lujinxingiaceae* fam. nov., were proposed. The genus *Lujinxingia* has been reclassified to the latter [51].

### 3.5. Order Herpetosiphonales in the Phylum Chloroflexota

Predatory species of the phylum *Chloroflexota* are known in the genus *Herpetosiphon* established in 1968 during the description of the filamentous gliding bacterium, *Herpetosiphon aurantiacus* gen. et sp. n. (=gen. nov., sp. nov.) [52]. One of the original strains used for the description was isolated from the slimy coating of *Chara* sp., a charophyte green alga, growing in Birch Lake, Minnesota, which implies predation on the slime-forming bacteria. The genus *Herpetosiphon* is placed in the family *Herpetosiphonaceae* within the order *Herpetosiphonales*, whose detailed descriptions were given by Gupta et al. (2013) [53].

An environmental strain, Hp g472 (DSM 52871), was isolated from a sandy soil in the beach of Poel island, Germany, and described as *Herpetosiphon gulosus* sp. nov., whose specific epithet derives from its gluttony, i.e., strong predatory activity [54].

Another environmental strain, CA052B, represents the fifth species of the genus *Herpetosiphonaceae*, described as *Herpetosiphon llansteffanense* sp. nov., whose specific epithet derives from the name of the origin, i.e., soil from the edge of a stream near the village of Llansteffan in Wales, UK [55]. This species demonstrates efficient predatory activity against a diverse array of prey microbial species using a “wolf pack” strategy that is possibly mediated by the secreted outer membrane vesicles containing a variety of hydrolytic enzymes [56].

### 3.6. Family Chitinophagaceae in the Phylum Bacteroidota

Biological soil crusts, or biocrusts, are regarded as promising mitigants for arid and semiarid lands. Filamentous cyanobacteria such as *Microcoleus* spp. play major roles in biocrust formation by stabilizing soil particles [57] and performing nitrogen fixation as well as carbon fixation via photosynthesis [58]. Biocrusts are applied to the restoration of damaged or disturbed lands [59]. The inoculation of “nursery grown” biocrusts have been attempted; however, inoculated biocrusts are often devastated by pathogen-like agents, leaving “plaques” [60]. From the diseased biocrusts, a cyanobacteria predator was enriched with *Microcoleus vaginatus* (PCC 9802), characterized by microscopy and physical–biochemical tests, genome-sequenced, and described as a new species, Ca. Cyanoraptor togatus. sp. nova., in the family *Chitinophagaceae* within the phylum *Bacteroidota* [61]. Bethany et al. (2022) [61] estimate that predation by Cyanoraptor reduces photosynthetic production of cyanobacteria by >10%.

### 3.7. Class Melainabacteria in the Phylum Cyanobacteria

Cyanobacteria can be predators, although they are preyed by predatory bacteria, such as Ca. *Cyanoraptor togatus*. However, the preys are not bacteria but eukaryotic microalgae such as *Chlorella*. The known predatory cyanobacterium is *Vampirovibrio chlorellavorus* [62] in the class *Melainabacteria* [63]. The members of the class *Melanibacteria* are affiliated with the phylum Cyanobacteria and characterized by nonphotosynthetic metabolisms and dark habitats, such as the human gut and groundwater [64]. Affiliation of the predatory *Vampirovibrio chlorellavorus* with the class *Melanibacteria* agrees with the nonphotosynthetic nature of this taxon. The genome of *Vampirovibrio chlorellavorus* was reconstructed in 2009 from the old co-culture with *Chlorella vulgaris* deposited in 1978 and dealt with in an academic article in 2015 [65]. There have been established strategies to defend commercially produced *Chlorella* from predation by *Vampirovibrio chlorellavorus* [66].

## 4. Phylogenetic Tree of Predatory Bacteria

**Figure 1:**
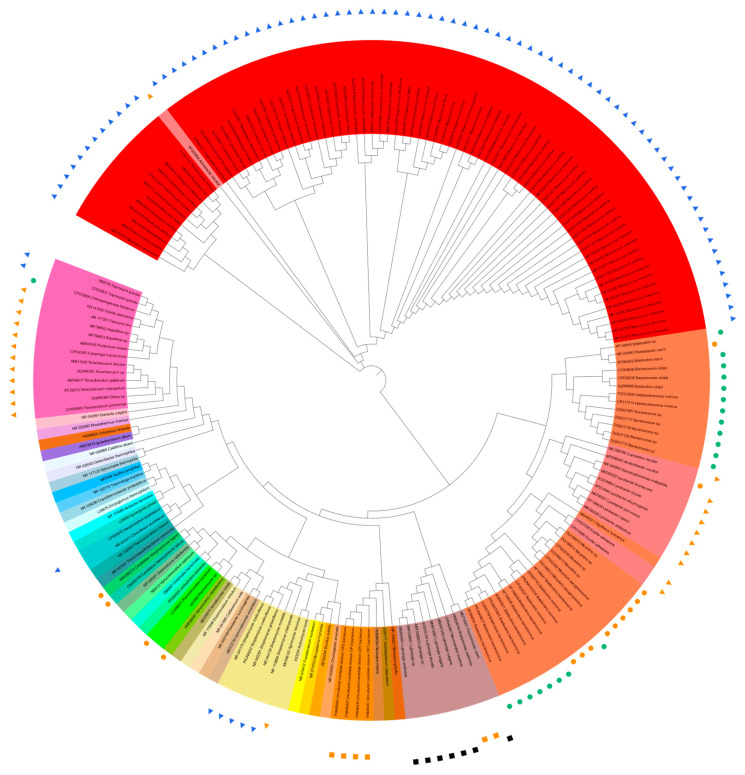

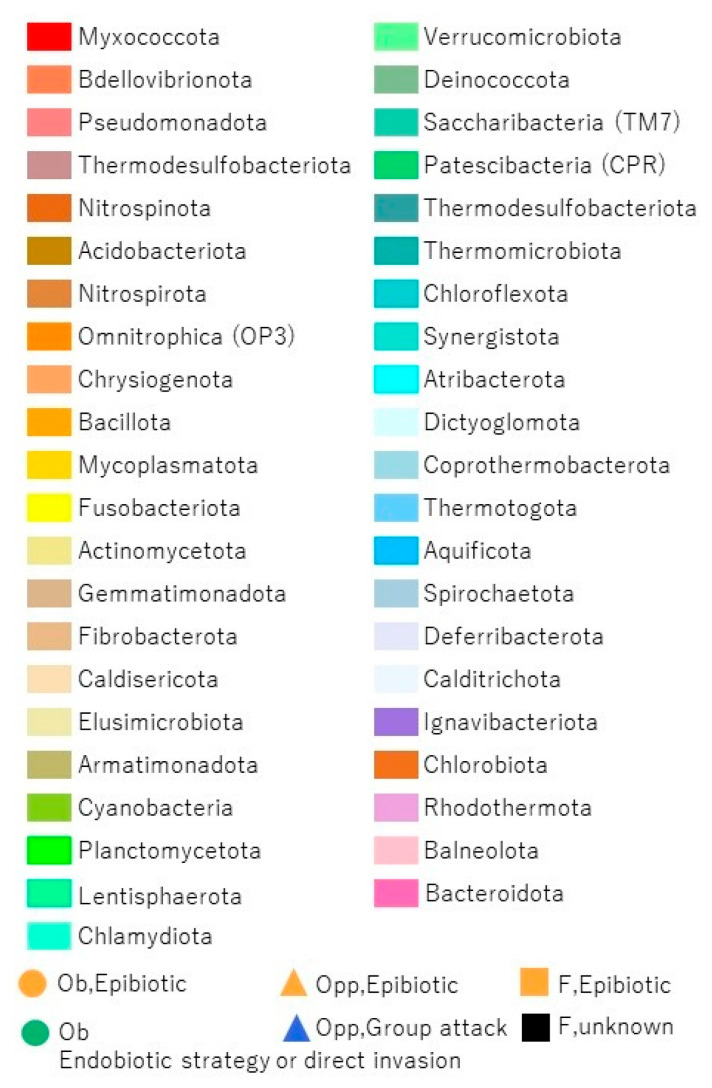
Trans-phylum phylogenetic tree of 135 sequences of predatory bacterial 16S rRNA genes listed in Table S1 [4,18,20,31,37,42,47,49,50,61,69,70,71,72,73,74,75,76,77,78,79,80,81,82,83,84,85,86,87,88,89,90,91,92,93,94,95,96,97,98,99,100,101,102,103,104,105,106,107,108,109,110,111,112,113,114,115,116,117,118,119,120], except [37] and 35 sequences from representative, current, and former bacterial phyla listed in Table S2 [121,122,123,124,125,126,127,128,129,130,131,132,133,134,135,136,137,138,139,140,141,142,143,144,145,146,147,148,149,150,151,152,153,154]. Predation properties are indicated by the symbols as follows: ●, obligate, epibiotic; ●, obligate, endobiotic or direct invasion; ▲, opportunistic, epibiotic; ▲, opportunistic, group attack; ■, facultative, epibiotic; and, ■, facultative, unknown.

A total of 136 sequences of predatory bacterial 16S rRNA genes were collected from 12 phyla (including candidate phyla) of *Actinobacteriota*, *Bacteroidota*, *Bdellovibrionota*, *Chloroflexota*, *Cyanobacteria*, *Desulfobacteriota*, *Myxococcota*, *Ca. Omnitrophica* (OP3), *Ca. Patescibacteria* (CPR) or Ca. Absconditabacteria (SR1), *Planctomycetota*, *Pseudomonadota*, and Ca. *Saccharibacteria* (TM7). The available 16S rRNA gene sequence of “*Ca. Vampirococcus lugosii*” (accession number MW286273, 1071 bp) [37] was the shortest among the collected sequences, and the phylogenetic trees with and without “*Ca. V. lugosii*” were constructed, along with the 35 reference sequences from current and former bacterial phyla. The sequences were aligned online with MEGA11 (https://www.megasoftware.net/; accessed on 20 May 2023) [67], and the phylogenetic trees based on the maximum likelihood method were drawn online with iTOL v6 (https://itol.embl.de/; accessed on 20 May 2023) [68] (Figure 1 and Figure S1). Figure 1 shows the phylogenetic tree based on the sequences of about 1.6 kb after alignment excluding the shortest 1071 bp sequence of “*Ca*. *V. lugosii*”. The 1.6 kb length, instead of the generally cited 1.5 kb, resulted from the alignment of 135 (136 minus 1, *Ca. V. lugosii*) full-length and near-full-length sequences that contain “gaps”.

Figure S1 displays the tree based on sequences of about 0.6 kb including “*Ca. V. lugosii*”. Information about the used 16S rRNA sequences of predatory bacteria [4,18,20,31,37,42,47,49,50,61,69,70,71,72,73,74,75,76,77,78,79,80,81,82,83,84,85,86,87,88,89,90,91,92,93,94,95,96,97,98,99,100,101,102,103,104,105,106,107,108,109,110,111,112,113,114,115,116,117,118,119,120] are listed in Table S1 along with the hunting strategies of the corresponding predators. The reference 16S rRNA gene sequences from 35 representative, current, and former bacterial phyla [121,122,123,124,125,126,127,128,129,130,131,132,133,134,135,136,137,138,139,140,141,142,143,144,145,146,147,148,149,150,151,152,153,154] are listed in Table S2.

The tree based on “1.6 kb-long” 16S rRNA gene sequences (Figure 1) shows (1) rather nonstreamlined phylogeny and predation strategies in the *Bdellovibrionota* (orange) and *Pseudomonadota* (pale pink) cluster from about 3:15 to 5:25 when Figure 1 is seen as the disk display of a 12 h clock and (2) streamlined phylogeny and predation strategies in the *Myxococcata* cluster (red), except the betaproteobacterial “AF005994 *Aristabacter necator*” [113] at 10:30. Interestingly, “AF005994 *Aristabacter necator*” was very deep-branched at about 10 o’clock on the “0.6 kb-long 16S-tree”, as shown in Figure S1.

Figure S1 (0.6 kb long 16S tree) includes the shortest sequence of “MW286273 *Vampirococcus lugosii*” [37] at about 8:30, neighbored with “OM390184 *Nanosynbacter lyticus*” [31]. Different from Figure 1, Figure S1 placed the nonpredatory “NR_042149 *Fibrobacter succinogenes*” [140] clustered with “AB540021 *Oligoflexus tunisiensis*” [128], which is predicted to be predatory [3] but as yet unconfirmed (Nakai, pers. comm.). These inconsistencies may be a hint for hunting novel predatory bacteria.

Another notable irregularity is “CP075895 *Chitinophagaceae bacterium*” at 9:10 in Figure 1 and 9:05 in Figure S1. This bacterium, *Ca. Cyanoraptor togatus* LGM1 [61], is the only known obligatory predator within the phylum *Bacteroidota* and is the only known endobiotic invader outside the *Bdellovibrionota* (orange)–*Pseudomonadota* (pale pink) cluster from a little before 3:00 to about 5:25. It is also the first obligatory, intracellular predator of cyanobacteria.

Only some of the predatory species of the phyla *Bdelovibrionota* and *Myxococcota* are shown in Figure 1 and Figure S1, which would have exhibited more significant proportions of the phyla if all the predatory species were included. However, the importance of the phyla in the phylogeny of predatory bacteria is already explicit in the current Figure 1 and Figure S1 with only selected species.

A myxobacterial species, *Sorangium cellulosum*, is not a typical epibiotic or endobiotic predator but a cellulolytic consumer as suggested by its genome [109], and its strong “lytic” activity may be regarded as predatory [155]. No such “lytic predators” are included in Figure 1 and Figure S1.

## 5. Future Perspective

According to the mitochondrial endosymbiotic theory, the origin of mitochondria is regarded as the endosymbiosis of an ancestral alphaproteobacterial linage [156]. A popular idea about the mechanisms of mitochondria acquisition is the engulfment or phagocytosis of proto-mitochondria by proto-eukaryotes [157]. Asgard archaea are regarded as a paraphyletic lineage of proto-eukaryotes [158]. However, Asgard archaea are genomically predicted to be nonphagocytotic [159], although a cultured Asgard archaeon “*Ca. Prometheoarchaeum syntrophicum*” is hypothesized to entangle–engulf–endogenize aerobic bacteria as metabolic partners like proto-mitochondria [160]. In any case, the endogenized proto-mitochondria have to stay “undigested”, and a new idea arose from the study of predatory bacteria. That is, the proto-mitochondrion was not undigested food but an “attenuated predator” [79,161,162]. More detailed studies on the known alphaproteobacterial predators, such as *Ensifer adhaerens* [111,112,163], as well as the finding of novel alphaproteobacterial predators, will shed more light on the relationship between the origins of mitochondria and predatory bacteria.

Cell sizes of predatory bacteria would be another perspective. Predatory bacteria of the candidate phylum radiation (CPR) represent ultra-small predators. Flat and stacked cells of 500–600 nm diameters and 200–250 nm heights are observed with the epibiotic predator or “sucker”, *Ca. Vampirococcus lugosii* (phylum *Ca. Absconditabacteria* or candidate division SR1), of photosynthetic anoxic bacteria [37]. Coccoid cells of 200–300 nm diameters are seen for the facultative epibiotic predator *Ca. Velamenicoccus archeaovorus* (phylum *Ca. Omnitrophica* or OP3) of Bacteria and Archaea [30,110]. Coccoid cells of 200–300 nm diameters along with rod cells are found for the epibiont predator *Ca. Nanosynbacter lyticus* (phylum *Ca. Saccharibacteria* or TM7) of human oral *Actinomyces odontolyticus* [31,32]. A recent transcriptomic study suggests that the *N. lyticus*–*A. odontolyticus* relationship is symbiotic rather than predatory [164]. The occurrences of these small predators (symbionts, parasites, or pathogens) support the idea that the ultra-small cell sizes of the CPR bacteria are associated with the small genome sizes, leading to dependence on other prokaryotes with fully (or more) functional genomes and larger cells [165].

Cells of *Bdelovibrio*, the obligate invading/attenuating predators, are also as small as 0.2–0.5 μm × 0.5–2.5 μm (200–300 nm × 500–2500 nm), larger than the ultra-small CPR bacteria but significantly smaller than the prey cells [22]. In contrast to these small-sized predators, the flat, round, or oval cells of the engulfing bacterium *Ca. Uab amorphum* (phylum *Planctomycetota*) are as large as ~4–5 μm in diameter, reaching 10 μm in diameter after the engulfment of bacterial preys [47]. Spherical to ovoid cells of *Gemmata obscuriglobus*, the not-bacteria-but-protein engulfing bacterium [166] within the phylum *Planctomycetota*, are in the moderate size range of 1.4–3.0 μm × 0.5–3.0 μm [167]. The type species of the phylum *Planctomycetota* is *Planctomyces bekefii*, which represents a rare example of as-yet-uncultivated bacteria with validly published names but no known identity [168]. The isolation and cultivation of more *Planctomycetota* species may assist in elucidating the origin and function of engulfment (endocytosis, phagocytosis) for predation [169,170].

## Data Availability

All the source information of the used DNA sequences are available in Tables S1 and S2. The processed DNA sequences for the construction of the phylogenetic trees (Figure 1 and Figure S1) are available in Supplementary Data S1 to S8.

## Supplementary Materials

The following supporting information can be downloaded at: https://www.mdpi.com/article/10.3390/microorganisms11071673/s1, Figure S1: Trans-phylum phylogenetic tree based on 136 (about 0.6 kb) sequences of predatory bacterial 16S rRNA genes listed in Table S1 and 35 sequences from representative, current, and former bacterial phyla listed in Table S2; Table S1: List of 136 sources of 16S rRNA gene sequences and hunting strategies of predatory bacteria; Table S2: List of 35 sources of reference 16S rRNA gene sequences from representative, current, and former bacterial phyla; Data S1: FASTA of a total of 170 (about 1.6 kb) 16S rRNA gene sequences of predatory bacteria for Figure 1. Data S2: FASTA of 171 (about 0.6 kb) reference 16S rRNA gene sequences for Figure S1; Data S3: 170 (about 1.6 kb, aligned) 16S rRNA gene sequences for Figure 1; Data S4: 171 (about 0.6 kb, aligned) 16S rRNA gene sequences for Figure S1; Data S5: 170 (about 1.6 kb, trimmed) 16S rRNA gene sequences for Figure 1; Data S6: 171 (about 0.6 kb, trimmed) 16S rRNA gene sequences for Figure S1; Data S7: 170 (about 1.6 kb, for iTOL) 16S rRNA gene sequences for Figure 1; Data S8: 171 (about 0.6 kb, for iTOL) 16S rRNA gene sequences for Figure S1.
