# Supplementary material for: Phylogenetic Revisit to a Review on Predatory Bacteria
Source: Microorganisms. 2023 Jun 27;11(7):1673. doi: 10.3390/microorganisms11071673 (PMC10385382; doi:10.3390/microorganisms11071673)
Supplement: Supplementary file 1 [file microorganisms-11-01673-s001.zip › Figure-S1.v2.pdf]

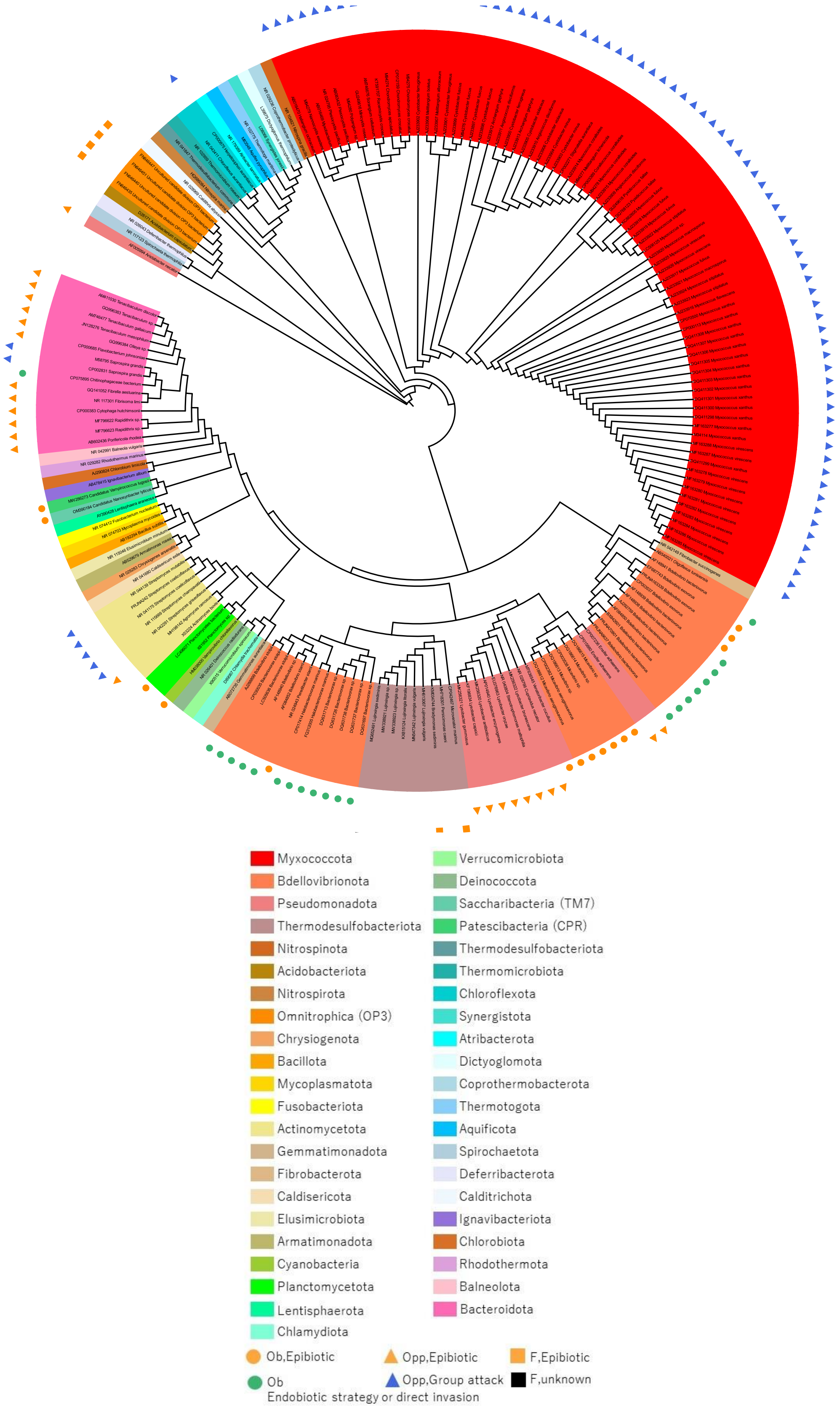

Figure S1. Trans-phylum phylogenetic tree of 135 (about 0.6 kb) sequences of predatory bacterial 16S rRNA genes listed in Table S1 [4,18,20,31,37,42,47,49,50,61,69-120] and 35 sequences from representative, current and former bacterial phyla listed in Table S2 [121-154]. Predation properties are indicated by the symbols as: ● obligate, epibiotic; ● obligate, endobiotic or direct invasion; ▲, opportunistic, epibiotic; ▲, opportunistic, group attack; ■, facultative, epibiotic; and, ■, facultative, unknown.

Figure S1 (0.6 kb-long 16S-tree) includes the shortest sequence of "MW286273 Vampirococcus lugosii" [37] at about 8:30, neighbored with "OM390184 Nanosynbacter lyticus" [31]. Different from Figure 1, Figure S1 placed the non-predatory "NR_042149 *Fibrobacter succinogenes*" [140] clustered with "AB540021 *Oligoflexus tunisiensis*" [128] that is predicted predatory [3] but yet-unconfirmed (Nakai, pers. comm.). These inconsistencies may be a hint for hunting novel predatory bacteria.
